# Study on Electrochemical Degradation of Nicosulfuron by IrO_2_-Based DSA Electrodes: Performance, Kinetics, and Degradation Mechanism

**DOI:** 10.3390/ijerph16030343

**Published:** 2019-01-26

**Authors:** Rui Zhao, Xuan Zhang, Fanli Chen, Xiaobing Man, Wenqiang Jiang

**Affiliations:** 1 College of Environmental Science and Engineering, Qilu University of Technology (Shandong Academy of Sciences), Ji’nan 250353, China; m13678823371@163.com (R.Z.); ahongjn@126.com (X.Z.); 2 Jinan Tianzheng Technology Co., Ltd., Ji’nan 250353, China; chenfanli@yeah.net; 3 Shandong Bluetown Analysis and Testing Co., Ltd, Ji’nan 250353, China; mxb0828@163.com

**Keywords:** nicosulfuron, electrochemical degradation, DSA electrode, degradation mechanism

## Abstract

The widely used sulfonylurea herbicides have caused negative effects on the environment and human beings. Electrochemical degradation has attracted much attention in the treatment of refractory organic compounds due to its advantage of producing no secondary pollution. Three kinds of IrO_2_-based dimensionally stable anodes (DSAs) were used to degrade nicosulfuron by a batch electrochemical process. The results showed that a well-distributed crack network was formed on the Ti/Ta_2_O_5_-IrO_2_ electrode and Ti/Ta_2_O_5_-SnO_2_-IrO_2_ electrode due to the different coefficients of thermal expansion between the Ti substrate and oxide coatings. The oxygen evolution potential (OEP) increased according to the order of Ti/RuO_2_-IrO_2_ < Ti/Ta_2_O_5_-SnO_2_-IrO_2_ < Ti/Ta_2_O_5_-IrO_2_. Among the three electrodes, the Ti/Ta_2_O_5_-IrO_2_ electrode showed the highest efficiency and was chosen as the experimental electrode. Single factor experiments were carried out to obtain the optimum electrolysis condition, shown as follows: currency intensity 0.8 A; electrode spacing 3 cm, electrolyte pH 3. Under the optimum conditions, the degradation of nicosulfuron followed first-order kinetics and was mainly due to indirect electrochemical oxidation. It was a typical diffusion-controlled electrochemical process. On the basis of the intermediate identified by high performance liquid chromatograph-mass spectrometry (HPLC-MS), two possible degradation routes were proposed.

## 1. Introduction

Sulfonylurea herbicides have been considered as “new formulation pesticides” because of their high selectivity and low persistence in the environment. They have been widely used to control a variety of broad-leafed weeds and grasses [1,2]. In recent years, with high toxicity and high residue herbicides fading out of the market, the production and application of sulfonylurea herbicides have been developing more rapidly. Their sales have accounted for more than 11% of the global herbicide market [3,4]. Sulfonylurea herbicides are highly soluble in water and have moderate to high mobility. Some of the commonly used sulfonylurea herbicides are able to remain in natural environmental medium such as soil and water [5,6]. The residual sulfonylurea herbicides can be transmitted through drinking water, the atmosphere, and plants, causing negative effects to the environment and human beings [7,8,9]. With China’s accession to the World Trade Organization, a large number of agricultural products have been exported to the United States, the European Union, and other regions. However, due to pesticide residues, many products are not able to meet the export standards. Therefore, how to remove the residues of sulfonylurea herbicides has become an urgent problem in China [10]. 

Nicosulfuron, as one kind of widely used sulfonylurea herbicide, is characterized by the presence of the pyridine and pyrimidine rings connected with a sulfonylurea bridge in the molecule. It is a selective systemic herbicide and can be absorbed by the foliage and roots of plants and rapidly transferred to the meristematic tissues in xylem and phloem [11]. Nicosulfuron is classified as being low to moderately persistent in soil, with maximum residual levels between 0.01 and 0.02 [12]. Despite its low persistence, its residues have been reported in many matrices, such as soil [13,14], surface water [15,16], and some crops [17,18]. 

Many research studies have been carried out on the degradation of nicosulfuron. Sabadie indicated that hydrolysis of nicosulfuron principally involved the breakdown of the urea part of the molecule at 30 °C at pH values ranging from 4 to 11. Its rate constants decreased from 0.50 d^−1^ to 0.002 d^−1^ as pH increased from 4 to 8, then remained stable under alkaline condition [19]. Lu et al. isolated a strain of *Aspergillus oryzae* whose degradation efficiency could reach 98.8% in a basic medium containing 2 mg/L of nicosulfuron [20]. Zhang et al. reported that a strain of *Serratia marcescens* N80 could degrade 93.6% nicosulfuron at a concentration of 10 mg/L in 96 h [21]. Song et al. isolated a strain of *Talaromyces flavus* from activated sludge in the wastewater treatment system of nicosulfuron manufacturer. Under optimum conditions (pH 6.1, 29 °C), it could degrade 100% of the initially added nicosulfuron (100 mg/L) within 5 days [22]. Researchers have isolated a variety of microorganisms from soil and other environmental media that are capable of degrading nicosulfuron. However, most of them had poor adaptability and slow degradation rates to high concentrations of nicosulfuron. They could not completely mineralize the target compound and the intermediate product(s) might be more toxic, which limited the practical application of biological treatments in nicosulfuron treatments [23].

Electrochemical oxidation, a kind of advanced oxidation process, can effectively avoid secondary pollution and is highly controllable. Also, it shows good degradation effects on refractory organics. Thus, electrochemical oxidation is considered to be a kind of environmentally friendly technology [24,25]. Electrode material is the most important dominant factor in realizing electrocatalytic processes. Good electrode material usually shows high stability, high conductivity, high catalytic activity, good selectivity, and low cost. 

Compared with metal electrodes, dimensionally stable anodes (DSAs), developed in the late 1960s and early 1970s, are less prone to creating pollutionand known as the “heart” of electrocatalytic oxidation [26]. The metal materials with strong corrosion resistance (such as Au, Pt, Ti, stainless steel, etc.) are used as the baseplate, and transition metal oxides, such as RuO_2_, IrO_2_, SnO_2_, TiO_2_, PbO_2_, MnO_2_, and Ta_2_O_5_, are used as coatings. The coating may be composed of one or more active metal oxides [27,28,29]. Due to the excellent performance of DSA electrodes, they have been extensively used in water electrolysis [30,31], the chlor-alkali industry [32,33], organic synthesis [34], and sewage treatment [35,36].

Up until present, studies on nicosulfuron degradation have mainly focused on biodegradation, especially at low concentrations. The electrochemical degradation of nicosulfuron has been less studied, and its electrochemical degradation mechanism is not yet clear.

In this paper, the effect and mechanism of nicosulfuron degradation by DSA electrode during electrochemical oxidation process were studied. Nicosulfuron was chosen as a model pollutant to explore three kinds of IrO_2_-based electrodes for their removal efficiency of toxic and recalcitrant organic compounds in aqueous solution. The surface morphology of the three IrO_2_-based electrodes were characterized by scanning electronic microscopy (SEM), linear sweep voltammetry (LSV), and cyclic voltammetry (CV) to select the best one. The selected electrode was used to study the electrochemical degradation of nicosulfuron. The effects of current intensity, electrolyte pH value, and electrode spacing on the degradation of nicosulfuron were investigated and the optimum condition was obtained. The degradation mechanism of nicosulfuron was proposed by identifying the intermediates. This experiment was expected to provide the theoretical basis and design ideas for the industrial design of the subsequent electrochemical degradation of nicosulfuron.

## 2. Experimental Materials and Methods

### 2.1. Experimental Materials

Nicosulfuron was obtained from Jingbo Agrochemicals Technology Co., LTD., Shandong, China, and used directly without any further purification. Its structural formula and general characteristics are shown in Table 1.

The methanol and acetic acid used in HPLC were of chromatographic grade. The other chemicals were of analytical grade and used without further purification. Solution was prepared using deionized water with the resistivity near to 18.3 MΩ⋅cm.

Three kinds of IrO_2_-based electrodes (Ti/RuO_2_-IrO_2_, Ti/Ta_2_O_5_-IrO_2_, Ti/Ta_2_O_5_-SnO_2_-IrO_2_) were provided by Suzhou Shude Industrial Machinery Co., Ltd., Jiangsu. Their sizes were 50 mm × 100 mm. The cathode was an electrode for titanium (Ti) with the same size. 

### 2.2. Analytical Methods

The surface morphology and composition of the electrodes were characterized by a SEM (Regulus8220, Hitachi, Japan) and x-ray energy dispersive spectrometer (EDS, QUANTAX, Bruker, Germany). The electrochemical performance of the IrO_2_-based electrodes was executed using LSV and CV at room temperature. A computer-controlled electrochemical workstation (CHI 660C, Shanghai Chenhua, China) with a conventional three-electrode cell system was used. The fabricated electrode (5 mm × 5 mm) was used as the working electrode, and a platinum sheet (5 mm × 5 mm) was used as the counter electrode. The saturated calomel electrode (SCE) was employed as the reference electrode. Before each experiment, it was cleaned by flame annealing and quenching with pure water [33].

The concentrations of nicosulfuron were measured with a HPLC (LC-10ATvp, SHIMADZU Corporation, Japan), using a mobile phase of methanol/water/acetic acid of 60:40:2 (V/V/V). Before the analysis, the mobile phase was sonicated for 30 min to remove the dissolved gas. The separation was performed using an InertSustain C18 column (150 mm × 4.6 mm × 5 μm, SHIMADZU Corporation, Tokyo, Japan) at the flow rate of 0.8 mL/min and column temperature of 303 K. The detection wavelength was 254 nm. 

The intermediate products of nicosulfuron degradation were identified by HPLC-MS (Agilent 6520B, Agilent Technologies, Santa Clara, CA, USA), equipped with an electrospray ionization source (ESI). As for the HPLC condition, methanol/water/acetic acid (60:40:2) was used as mobile phase and flow rate was set to 0.4 mL/min with a Shim-pack VP-ODS liquid chromatography column (150 mm × 4.6 mm × 5 μm, SHIMADZU Corporation, Tokyo, Japan). The injection volume was 1 μL and the column temperature was 303 K. The MS conditions were in positive ion detection mode and mass range was between 50 m/z and 1600 m/z. The following parameters were used: ion source temperature 373 K, dry gas flow rate 10 L/min, atomizer pressure 35 psig, capillary voltage 3500 V, capillary outlet voltage 120 V, cone voltage 65 V, and eight-pole RF voltage 750 V.

### 2.3. Electrolysis Experiment

**Figure 1:**
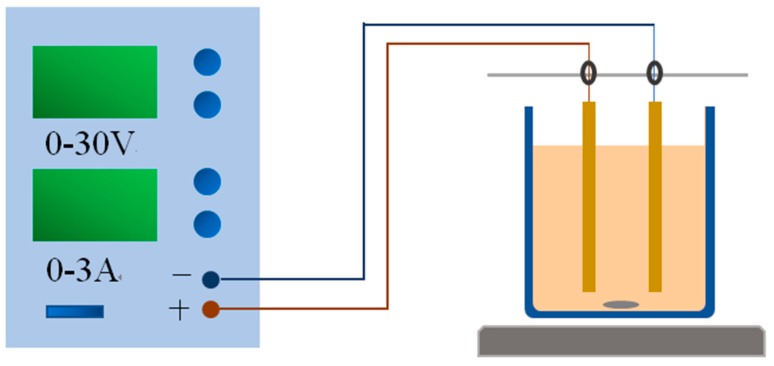
Electrolysis setup.

Electrolysis experiments were performed by batch processes under galvanostatic condition to study the electrocatalytic performance of the electrodes (Figure 1). The IrO_2_-based electrodes were used as the anode accompanied with a Ti electrode of the same size as the cathode. To examine the influencing parameters, a volume of 400 mL nicosulfuron solution was used to conduct the electrolysis experiments. The reaction conditions included current intensity (0.3 A, 0.5 A, 0.8 A, and 1.0 A), pH value (1.0, 3.0, 6.0, and 9.0), and electrode spacing (1.5 cm, 3 cm, 4.5 cm, and 6 cm). For the supporting electrode, 0.1 M Na_2_SO_4_ was used. During the experiments, samples were drawn from the reactor at certain time intervals. The concentration of nicosulfuron was quantitatively analyzed by HPLC. Each experiment was conducted three times. The results used the average values and the relative standard deviations (RSDs) were less than 5%.

## 3. Results and Discussion

### 3.1. Characterization of IrO_2_-Based Electrodes

#### 3.1.1. Structure and Morphology of the Electrode Coatings

**Figure 2:**
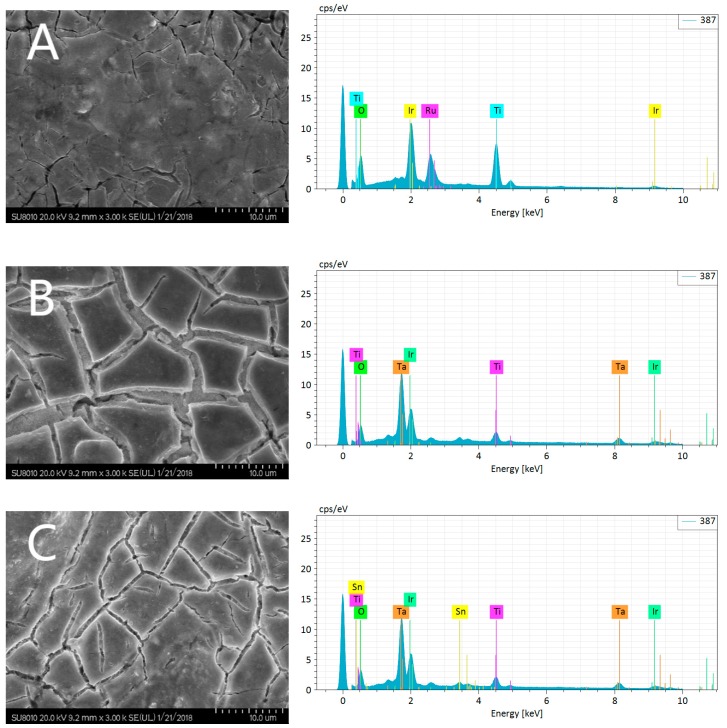
SEM images and energy dispersive spectrometer (EDS) spectra of Ti/RuO_2_-IrO_2_ (**A**), Ti/Ta_2_O_5_-IrO_2_, (**B**) and Ti/Ta_2_O_5_-SnO_2_-IrO_2_ (**C**).

The SEM micrographs of the IrO_2_-based electrodes as well as their EDS elemental analysis are shown in Figure 2. The three kinds of IrO_2_-based electrodes all showed “cracked” structures, which was a typical feature of oxide electrodes formed during heat-expansion and cold-contraction processes. The formation of the crack was due to the different coefficients of thermal expansion between the Ti substrate and oxide coatings [37,38]. The cracks connected with each other as networks. Especially, the networks were well-regulated and well-distributed on the surface of the Ti/Ta_2_O_5_-IrO_2_ electrode and Ti/Ta_2_O_5_-SnO_2_-IrO_2_ electrode, which helped to increase the real surface area of the electrode. Their corresponding EDS spectra displayed clear oxygen and iridium peaks for each electrode. The distinct additive metal peak was present for each of the binary oxide coatings. Further quantitative analysis showed that the metal oxide for each binary composition was present in approximately 20% wt.

#### 3.1.2. Linear Sweep Voltammetry Measurement (LSV)

**Figure 3:**
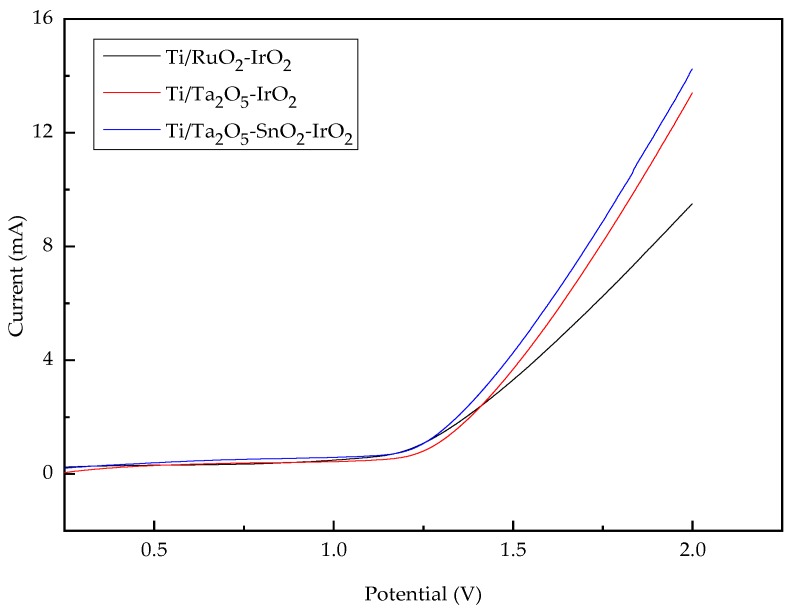
Linear sweep voltammetry (LSV) curves of three electrodes at scan rate of 50 mV/s.

The oxygen evolution potential (OEP) can characterize the difficulty of the oxygen evolution reaction on the electrodes. A high OEP is advantageous for restraining the occurrence of side reactions, enhancing the organics degradation ability and weakening the influence of oxygen to peel the catalyst layer. Therefore, the OEP is positively correlated with the organics degradation ability of the anodes [39]. The performance of the electrodes varied with the baseplate composition and preparation method of the oxide film. Higher oxygen evolution potential can be obtained by improving the material and structure of electrodes [40,41]. In this paper, LSV curves were used to examine the OEPs of the three electrodes. As shown in Figure 3, the OEPs increased according to the order of Ti/RuO_2_-IrO_2_ < Ti/Ta_2_O_5_-SnO_2_-IrO_2_ < Ti/Ta_2_O_5_-IrO_2_. The Ti/Ta_2_O_5_-IrO_2_ electrode with higher OEP should have better degradation performance and lower power consumption during the electrochemical degradation process.

#### 3.1.3. Cyclic Voltammetry (CV)

**Figure 4:**
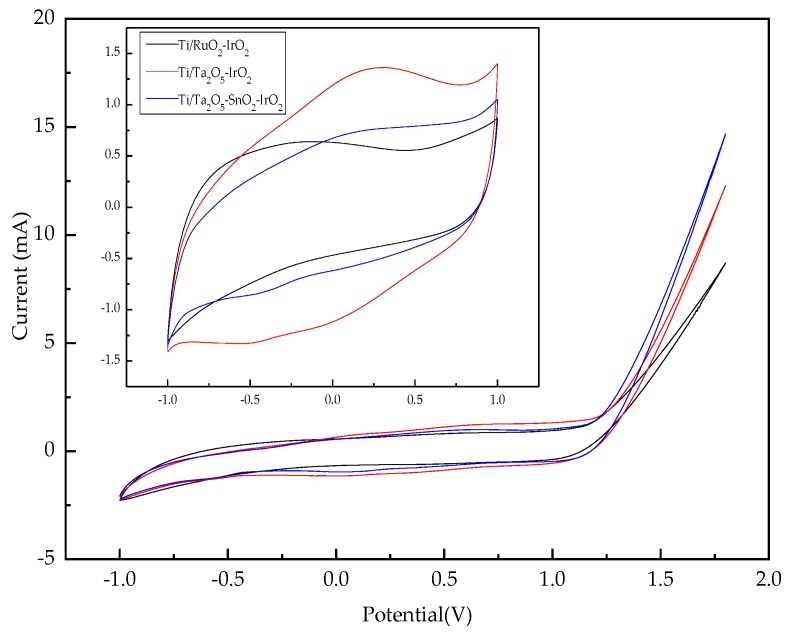
Cyclic voltammetry (CV) curves of three electrodes at a scan rate of 50 mV/s.

Figure 4 shows typical CV curves of three IrO_2_-based electrodes in the long potential range between −1.0 V and +1.8 V at a scan rate of 50 mV/s. The shape indicates that their initial OEP was about +1.3 V. When the applied potential was higher than +1.3 V, the current increased with increasing electrode potential. Thus, the CV curves were measured between −0.1 V and +1.0 V in the short potential region. The electrochemical effective surface areas of the three IrO_2_-based electrodes were estimated based on the CV curves recorded in the short potential, revealing that the electrochemical active surface areas reduced in the following order: Ti/Ta_2_O_5_-IrO_2_ > Ti/Ta_2_O_5_-SnO_2_-IrO_2_ > Ti/RuO_2_-IrO_2_. This was consistent with the SEM images shown in Figure 2.

### 3.2. The Effect of Electrode Material

**Figure 5:**
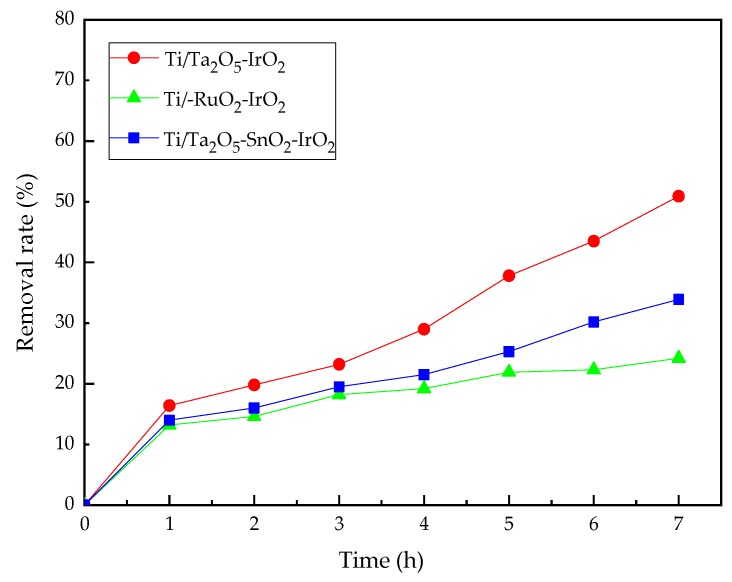
Effect of different electrodes on the degradation of nicosulfuron.

Electrode materials are the most important part of electrochemical oxidation reactors. Therefore, the development of electrodes with good performance and high stability has become a major research focus [42]. Three kinds of IrO_2_-based electrodes were used as anodes to degrade nicosulfuron under the condition of current intensity 0.3 A, electrodes spacing 3 cm, and pH 6. The effects of different electrodes on the removal rate are shown in Figure 5. 

Ti electrodes coated with transient metal oxides have been studied extensively due to their excellent electrocatalytic activities [43]. Electrodes with different coatings exhibit different electrocatalytic properties. As shown in the Figure 5, the catalytic performance of the Ti/Ta_2_O_5_-IrO_2_ electrode was better than that of the two other electrodes. The reason might be that the Ti/Ta_2_O_5_-IrO_2_ electrode could provide a larger surface area and higher OEP for the electrochemical reaction. In the following experiment, the Ti/Ta_2_O_5_-IrO_2_ electrode was chosen as the experimental electrode.

### 3.3. The Effect of Current Intensity

Current intensity is an important experimental parameter that affects the electrochemical oxidation process, as it determines the production capacity of hydroxyl radicals (·OH s) on the electrode, which decides the removal rate of organic matter in aqueous solutions [44]. However, the higher the current intensity is, the greater the power consumption is. Thus, it is extremely essential to choose an appropriate current intensity to achieve a high electrolysis efficiency along with a low energy consumption. A suitable current intensity is essential for achieving high electrolysis efficiency with low power consumption.

**Figure 6:**
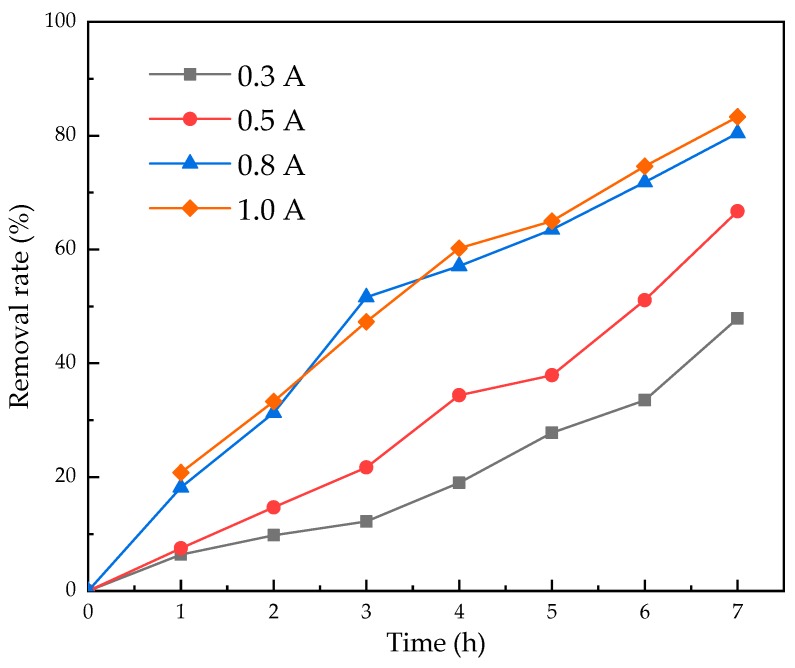
Effect of current intensity on the electrolysis of nicosulfuron.

The effect of current intensity on nicosulfuron electrolysis is shown in Figure 6. The increase of current intensity had a positive influence on the degradation of nicosulfuron in the range of 0.3–1.0 A. When the current intensity was 0.8 A, nicosulfuron removal rate reached 83.3% after electrolysis for 7 h, which was almost the same as that of 1.0 A. The increase of current intensity had dual effects on electrolysis. The high current intensity helps to speed up the production rate of ·OH and then accelerate the oxidation rate of organic matter on the electrode surface [45]. However, the excessive current intensity was conducive to the occurrence of side reactions, consuming the formed ·OH and reducing its availability. The current intensity was too low to meet the energy requirement for electrolysis. The selected optimal current intensity in this experiment was 0.8 A.

### 3.4. The Effect of Electrode Spacing

**Figure 7:**
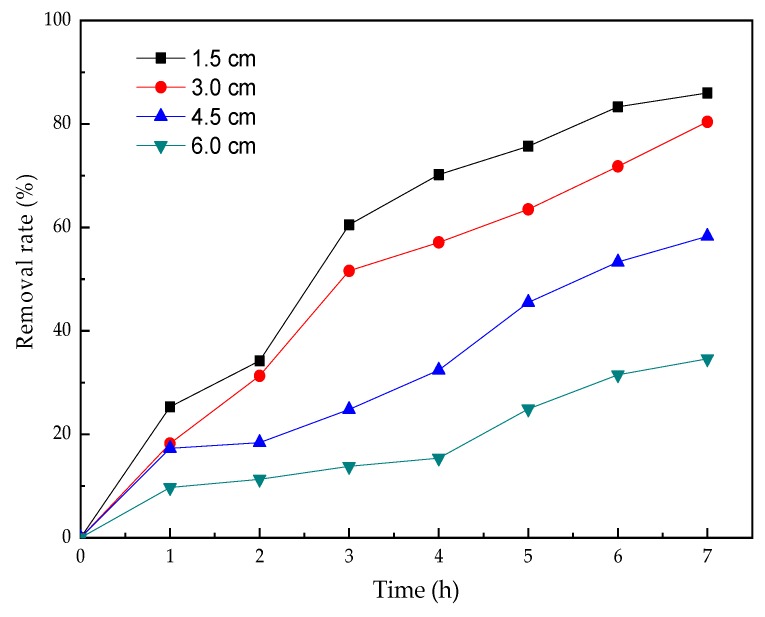
Effect of electrode spacing on the electrolysis of nicosulfuron.

Electrolytic experiments were performed at different spacings to study the influence on nicosulfuron degradation and results are shown in Figure 7. When the electrode spacing was 1.5 cm, the highest removal rate was obtained. In the same electrolyte system, the smaller the electrode spacing was, the shorter the distance the electrons were required to transfer, which was advantageous in terms of increasing energy transfer during the electrolysis process. Therefore, the short distance was helpful for increasing the electrolysis efficiency [19]. However, the excessively small electrode spacing would lead to a low treating volume between the electrodes, which was not beneficial to improving treatment efficiency. Herein, the electrode spacing of 3 cm was chosen for the rest of the experiments.

### 3.5. The Effect of Electrolyte pH

**Figure 8:**
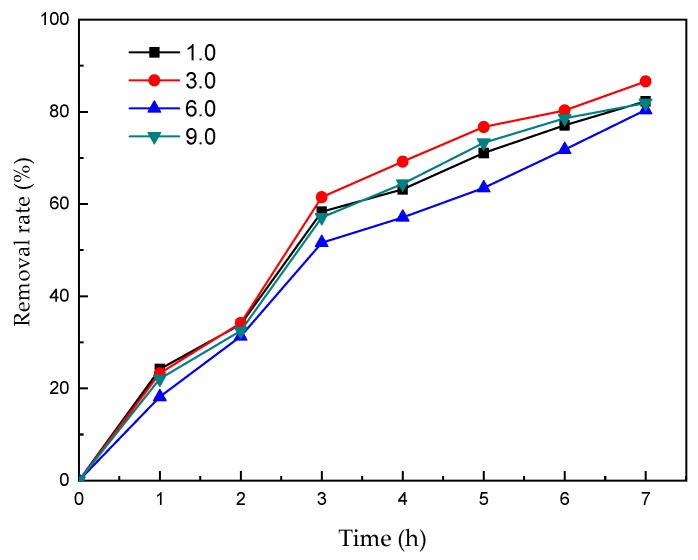
Effect of pH on the degradation of nicosulfuron.

Solution pH is one of the most important factors that affects the performance of electrochemical processes. The effect of pH values ranging from 1 to 9 on the nicosulfuron degradation was investigated, and the results are presented in Figure 8. The removal rate of nicosulfuron was higher in acid conditions or in alkaline conditions than in neutral conditions. The maximum removal rates were obtained at pH 3, which was 86.6% at 7 h. The optimum reaction pH was 3.0 for nicosulfuron degradation.

Zhao et al. indicated that nicosulfuron could be degraded to some extent via chemical hydrolysis under natural conditions, especially under acidic pH conditions [46]. The low pH inhibited the oxygen evolution reaction and resulted in the improvement of nicosulfuron degradation efficiency [47]. This result was in agreement with that of Ruan et al. [48], who proved that the degradation rates of sulfonylurea herbicides were affected by pH and that acidic pH was beneficial to the degradation process.

### 3.6. Optimization Experiment

**Figure 9:**
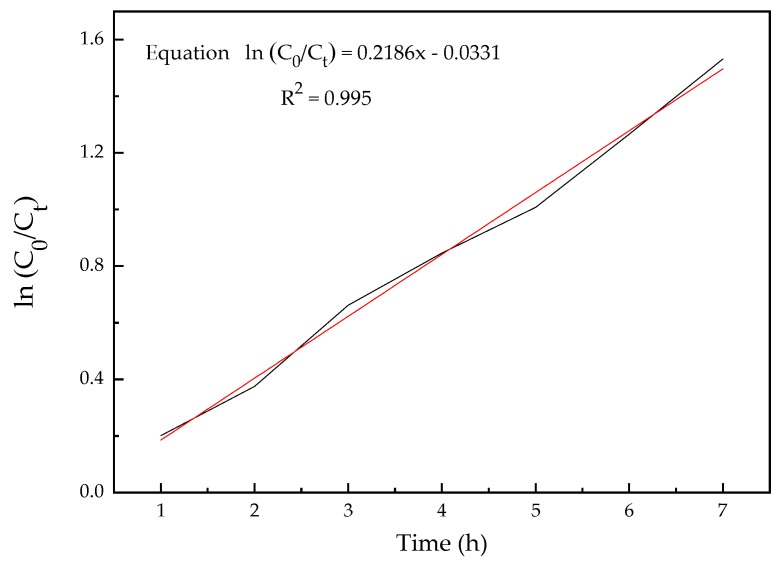
Fitting kinetic curve under optimal electrolysis conditions.

According to the results of the single factor experiments conducted above, the optimum conditions were obtained as follows: current intensity 0.8 A, electrode spacing 3 cm, and pH 3. An electrolysis experiment was conducted under the optimum conditions, and the variation of nicosulfuron concentration with time is shown in Figure 9. A kinetic equation was obtained by the least-square method, which is shown in Equation (1).

(1) $$\ln(C_{0}/C_{t})=0.2186t-0.0331$$

where *C*_0_ was the initial concentration of nicosulfuron, and *C_t_* was the concentration of nicosulfuron at a given time (*t*). The equation indicated that the degradation of nicosulfuron under optimum conditions followed first-order kinetics.

Under the optimum conditions, the removal rate of nicosulfuron at 100 mg/L could exceed 80% by 7 h electrolysis, which indicated that electrochemical processes could complete the degradation process in a relatively short time. In comparison, biological treatments usually have taken several days to achieve more than an 80% removal rate. Zhao et al indicated that the strain ZWS11 adapted to the initial concentrations as high as 500 mg/L and degraded about 80% at initial concentration levels of 5–500 mg/L in 6 days [46]. Qi et al. suggested that *B. subtilis* could degrade approximately 80% of nicosulfuron at the initial concentration of 400 mg/L within 3 days [49]. Rapid degradation of nicosulfuron has been considered to be an urgent issue for controlling nicosulfuron contamination. The electrochemical method, as an advanced oxidation process, could provide suitable conditions for the rapid degradation of nicosulfuron. 

### 3.7. Electrochemical Degradation Mechanism of Nicosulfuron on Ti/Ta_2_O_5_-IrO_2_ Electrode

#### 3.7.1. Electrochemical Mechanism

**Figure 10:**
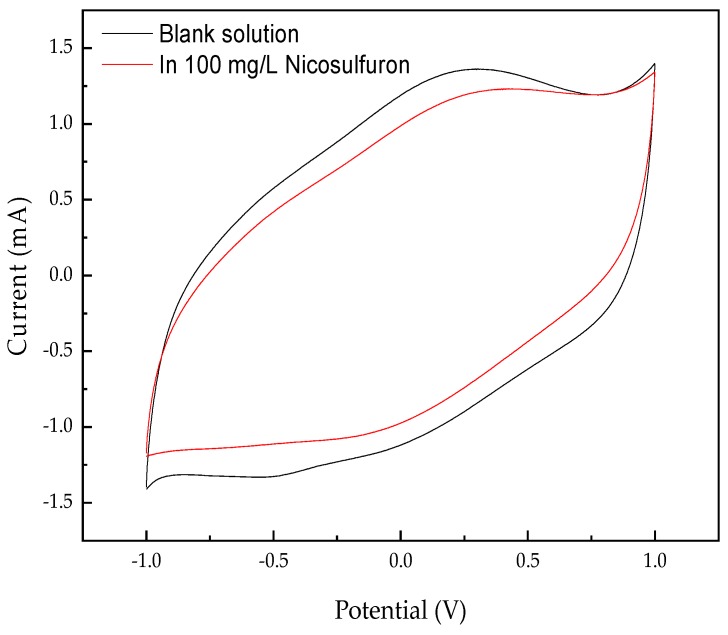
CV curves of the Ti/Ta_2_O_5_-IrO_2_ electrode in the absence and in the presence of nicosulfuron.

The Ti/Ta_2_O_5_-IrO_2_ electrode was characterized by CV tests in 0.1 M Na_2_SO_4_ solution in the absence and in the presence of 100 mg/L nicosulfuron. Compared with that in the blank solution (Figure 10), no additional peak was found in the 100 mg/L nicosulfuron solution, which indicated that the direct electron transfer did not occur on the Ti/Ta_2_O_5_-IrO_2_ electrode. The electrochemical oxidation of organics on the surface of the anode was divided into direct oxidation and indirect oxidation. If there was no new anodic current peak during the positive sweep process, it meant that oxidization was not occurring directly on the surface of the anodes and the degradation of organics was mainly carried out by indirect oxidation. Thus, in this experiment, nicosulfuron was not oxidized directly on the surface of the anode but was decomposed by the oxidizing agent that existed in the solution, such as the hydroxyl radical (·OH). Equations (2) and (3) indicate the indirect oxidation process. MO_x_ represents the metal oxide coating of Ti/Ta_2_O_5_-IrO_2_ electrode.

(2) $$MO_{x}+H_{2}O\to MO_{x}(\cdot \mathrm{OH})+H^{+}+e^{-}$$

(3) $$MO_{x}(\cdot \mathrm{OH})\to MO_{x}+O_{2}+2H^{+}+2e^{-}$$

**Figure 11:**
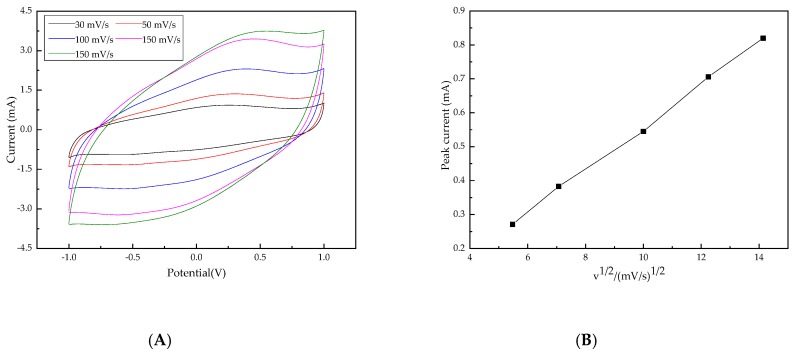
CV curves of the Ti/Ta_2_O_5_-IrO_2_ electrode at different scan rates. (**A**) CV curves at different scan rates; (**B**) The plots of peak current vs. the square root of scan rate.

The voltammetric response on the Ti/Ta_2_O_5_-IrO_2_ electrode at different scan rates is shown in Figure 11a. The CV curves at all scan rates remained closed, indicating that surface charge had enough time to access the entire solution at both high and low scan rates [50]. Due to pseudo-capacitive behavior, redox peaks were seen in the CV curves [51]. The redox couple in all CV curves exhibited an increased current response with the increase of the scan rate from 10 to 150 mV/s. It showed that the peak currents at different scan rates were directly proportional to the square root of the scan rates (v½) (Figure 11b), indicating that the redox reactions on the Ti/Ta_2_O_5_-IrO_2_ electrode were a typical diffusion-controlled electrochemical process [52,53].

#### 3.7.2. Electrochemical Degradation Mechanism of Nicosulfuron

Many studies have proved that the new intermediate products, formed during the degradation process of nicosulfuron, might be more toxic and stable than the precursor herbicides [1,54,55]. Thus, it is necessary to identify the intermediate products and investigate their toxicity to the environment.

Nicosulfuron was electrolyzed under the optimum condition of current intensity 0.8 A, electrode spacing 3 cm, and pH 3, and the sample solution with the electrolysis removal rate of about 50% was subjected to HPLC-MS detection. Three peaks were obtained by LC separation, and their retention times were 4.767~4.850 min, 6.217~6.367 min, and 7.900~8.000 min, respectively. 

**Figure 12:**
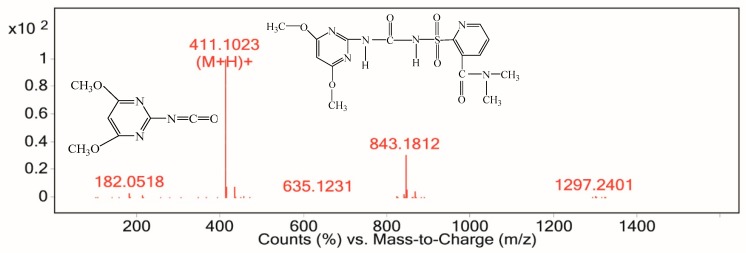
Electrospray ionization source (ESI) spectrum from 6.217 min to 6.367 min.

**Figure 13:**
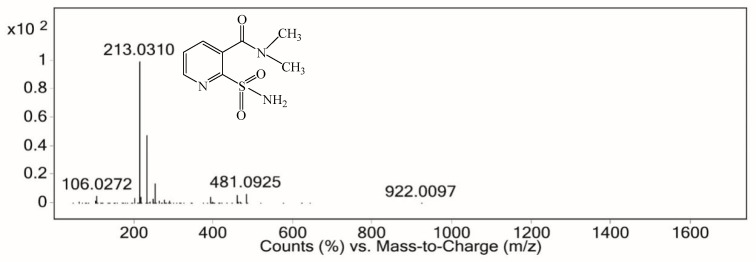
ESI spectrum from 4.767 min to 4.850 min.

**Figure 14:**
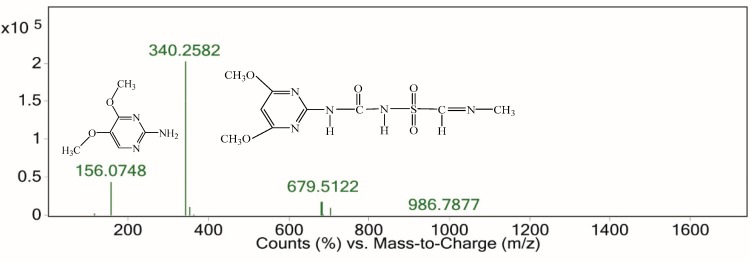
ESI spectrum from 7.192 min to 7.309 min.

According to the chemical structure and reported degradation pathway of nicosulfuron, the detected fragments were deduced and shown in Figure 12, Figure 13 and Figure 14. The ESI analysis showed the presence of the pseudo-molecular ion at m/z 411 ([M+H]^+^), and the speculative substance was nicosulfuron (C_15_H_18_N_6_O_6_S). The signal at m/z 843 was attributed to the sodium adduct of the dimeric form of the pseudo-molecular ion ([2M+Na]^+^) (Figure 12).

The intermediate product with retention time from 4.767 min to 4.850 min gave a molecular ion at m/z 213 ([M+H]^+^), derived from the cleavage of the C–N bond of sulfonylureic bridge and from the loss of the N–CH_2_–CH_3_ group. It was identified as 2-aminosulfonyl-N,N-dimethylnicotinamide (C_8_H_11_N_3_O_3_S)(Figure 13) and was considered to be the main product of nicosulfuron electrolysis. 

The intermediate product with retention time from 7.192 min to 7.309 min gave a fragment ion at m/z 156, which was an indication of ([M+H]^+^). The speculative substance was identified as 4,5-dimethoxypyrimidin-2-amine (C_6_H_9_N_3_O_2_) (Figure 14). The other fragment at m/z 340 was derived from the opening of the pyridinic ring. The m/z 182 fragment (Figure 12) was derived from the cleavage of the C–N bond of the sulfonylureic bridge.

**Figure 15:**
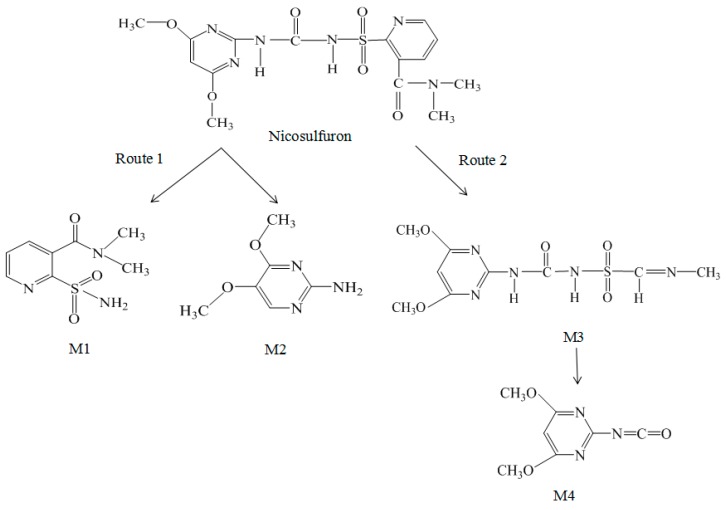
Degradation mode under the conditions of nicosulfuron electrolysis.

From the speculated degradation product information, two degradation pathways of nicosulfuron under the electrolysis conditions were proposed, as shown in Figure 15. Route 1 was the cleavage of the C–N bond of the sulfonylureic bridge and produced M1 and M2, which were the two most important intermediates during the nicosulfuron synthesis process. M1 and M2 have also been reported as degradation products by microorganisms [10,22,44]. Sabadie reported that the hydrolysis of nicosulfuron under acidic conditions was also carried out in accordance with route 1 [19]. Route 2 was the opening of the pyridinic ring and formed M3, which was also found by Benzi et al. during the photoinduced gradation process using a xenon lamp [1]. M4 was produced through the further cleavage of the C-N bond in the side chain of M3. The different results were due to the different reactive conditions, indicating that the reactive condition would influence the degradation pathway of nicosulfuron [19]. 

## 4. Conclusions

The electrochemical method was chosen to degrade nicosulfuron due to its controllability and environmental friendliness. Three kinds of IrO_2_-based DSA electrodes were used to electrolyze nicosulfuron by a batch process. A well-distributed crack network was formed on the Ti/Ta_2_O_5_-IrO_2_ electrode and Ti/Ta_2_O_5_-SnO_2_-IrO_2_ electrode. Their OEPs increased according to the order of Ti/RuO_2_-IrO_2_ < Ti/Ta_2_O_5_-SnO_2_-IrO_2_ < Ti/Ta_2_O_5_-IrO_2_. Nicosulfuron was used as the model pollutant to choose the electrode with the highest performance. The Ti/Ta_2_O_5_-IrO_2_ electrode showed the highest efficiency and was chosen for use as the anode in the electrochemical experiment. The obtained optimum electrolysis conditions were: currency intensity 0.8 A, electrode spacing 3 cm, electrolyte pH 3. Under the optimum conditions, the degradation of nicosulfuron followed first-order kinetics and was mainly due to indirect oxidation. The redox reaction on the Ti/Ta_2_O_5_-IrO_2_ electrode was a typical diffusion-controlled electrochemical process. On the basis of the intermediates identified by HPLC-MS, two possible degradation routes of nicosulfuron were proposed, including the cleavage of the C–N bond of the sulfonylureic bridge and the opening of the pyridinic ring.

## Figures and Tables

**Table 1:** General characteristics of nicosulfuron.

Name	Nicosulfuron
Chemical structure	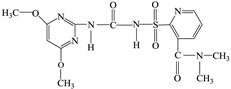
Molecular Formula	C_15_H_18_N_6_O_6_S
CAS Number	111991-09-4
Molecular mass	410.41 g/mol
Solubility	acetonitrile 2.3%, acetone 1.8%, ethanol 0.45%, water 12%

CAS: Chemical Abstracts Service.
